# Deviating glucose results in an international dual-center study. A root cause investigation

**DOI:** 10.11613/BM.2022.011001

**Published:** 2021-12-15

**Authors:** Janne Cadamuro, Peter Bergsten, Katharina Mörwald, Anders Forslund, Marie Dahlbom, Jonas Bergquist, Iris Ciba, Susanne M. Brunner, Jeanne Jabbour, Daniel Weghuber

**Affiliations:** 1 Department of Laboratory Medicine, Paracelsus Medical University, Salzburg, Austria; 2 Department of Medical Cell Biology, Uppsala University, Uppsala, Sweden; 3 Department of Women’s and Children’s Health, Uppsala University, Uppsala, Sweden; 4 Pediatric Gastroenterology, Hepatology and Nutrition, Department of Pediatrics, University Hospital of the Paracelsus Medical University, Salzburg, Austria; 5 Obesity Research Unit, University Hospital of the Paracelsus Medical University, Salzburg, Austria; 6 Analytical Chemistry and Neurochemistry, Department of Chemistry – Biomedical Center, Uppsala University, Uppsala, Sweden; 7 Department of Pediatrics, University Hospital of the Paracelsus Medical University, Salzburg, Austria

**Keywords:** preanalytical phase, tube additive, PREDICT, blood collection tubes, preanalytical error

## Abstract

During a dual-center study on obese and normal weight children and adolescents, focusing on glucose metabolism, we observed a marked difference in glucose results (N = 16,840) between the two sites, Salzburg, Austria and Uppsala, Sweden (P < 0.001). After excluding differences in patient characteristics between the two populations as cause of this finding, we investigated other preanalytic influences. Finally, only the tubes used for blood collection at the two sites were left to evaluate. While the Vacuette FC-Mix tube (Greiner Bio-One, Kremsmünster, Austria) was used in Uppsala, in Salzburg blood collections were performed with a lithium heparin tube (LH-Monovette, Sarstedt, Germany). To prove our hypothesis, we collected two blood samples in either of these tubes from 51 children (Salzburg N = 27, Uppsala N = 24) and compared the measured glucose results. Indeed, we found the suspected bias and calculated a correction formula, which significantly diminished the differences of glucose results between the two sites (*P* = 0.023). Our finding is in line with those of other studies and although this issue should be widely known, we feel that it is widely neglected, especially when comparing glucose concentrations across Europe, using large databases without any information on preanalytic sample handling.

## Introduction

The dual-center cross-sectional Beta-JUDO (Beta-cell function in JUvenile Diabetes and Obesity) study (FP7-HEALTH-2011-two-stage, project number: 279153) was carried out at Uppsala University Hospital, Sweden and Paracelsus Medical University Hospital in Salzburg, Austria. In the project well-characterized European patient cohorts of children and adolescents aged 6 to 18 years with obesity as well as normal weight control subjects were characterized with particular emphasis on insulin secretion and glucose metabolism.

To exclude any analytical differences, a validation round for the 38 analytes of interest was organised by the Uppsala University Hospital prior to the conduction of the study. For glucose comparison, dipotassium-ethylenediaminetetraacetic acid (K2-EDTA) samples from eight healthy fasting and non-fasting adult volunteers were collected on ice-water slurry, centrifuged at + 4 °C at 2500xg, aliquoted and frozen at - 70 °C. Always one part of these prepared aliquot pairs was then shipped on dry ice to the University Hospital of Salzburg for laboratory testing and the other was thawed and analysed in the Uppsala laboratory. Thus, preanalytical conditions were identical for all aliquot pairs.

After reaching a consensus about the comparability of analytical methods between the two laboratories, the study started and samples from 865 obese and normal-weight children (376 in Salzburg and 489 in Uppsala) were collected at several time-points in each individual.

Altogether, 16,840 glucose results, some from fasting patients, some collected during an oral glucose tolerance test (OGTT), were acquired during the study (Uppsala: N = 6250; Salzburg: N = 10,590). After excluding extreme values within these results (N = 79), identified by application of the 3.0 IQR rule, the Kolmogorov-Smirnov test showed a non-normally distributed data set. Therefore, the Mann-Whitney-U-test was applied to test for differences between the groups. A systematic difference in glucose concentrations between Swedish and Austrian children became evident (P < 0.001), with higher values in Uppsala samples. Several biasing variables, potentially explaining these differences, were statistically analysed by co-variant ANOVA and linear regression models, including age, gender, body mass index, family history of diabetes, and ethnic origin. There were significant differences in Body-Mass-Index Standard Deviation Score (BMI-SDS) between all groups, except between Salzburg controls and Uppsala controls. Mean fasting glucose concentrations in the Uppsala control cohort as well as in the Uppsala patient cohort were higher compared to both the Salzburg patient and control group. When including the fact that mean BMI-SDS was lower in Uppsala controls than in Salzburg patients, the findings led to the hypothesis that differences in mean BMI-SDS could not be cause for the observed shift. Thus, results from the validation round were reassessed, this time using the blood collection tube additives that had been used during the clinical part of the study at the different sites (K2-EDTA and lithium-heparin (LH) Na-F/C).

## Laboratory analyses

Study population characteristics was showed in Table 1.

**Table 1:** Study population characteristics

	**Salzburg**		**Uppsala**	
**Controls**	**Patients**	**Controls**	**Patients**
N	55	321	70	419
Gender, F (proportion)	22/55	161/321	32/70	180/419
Age (years)	16 (12-18)	13 (9-19)	13 (6-18)	13 (3-18)
BMI-SDS	0.36 ± 1.03	2.82 ± 0.61	0.06 ± 1.11	3.17 ± 0.62
Age is presented as median (range). F – female. BMI-SDS – Body Mass Index – Standard deviation score. BMI-SDS is presented as mean and standard deviation.				

**Figure 1:**
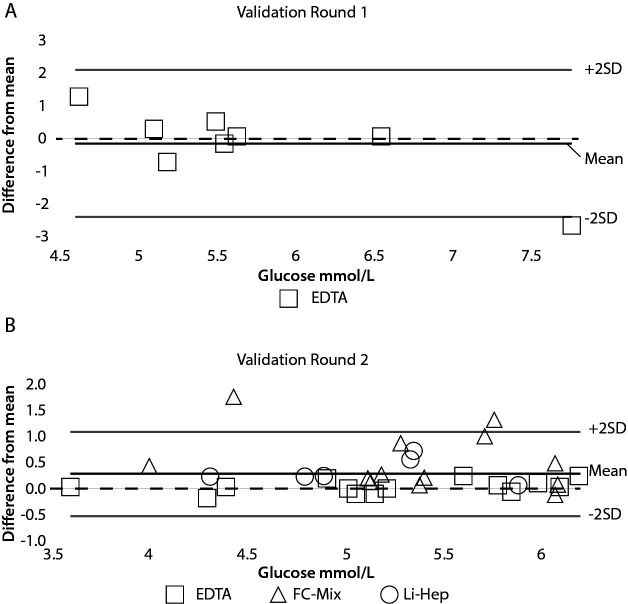
Results from the glucose testing during validation round 1 and 2. SD – standard deviation. EDTA – ethylenediaminetetraacetic acid. FC-Mix – Vacuette FC-Mix tube. Li-Hep – lithium heparin tube.

Results from the first and second validation rounds are presented in Figure 1.

## Further investigations

**Figure 2:**
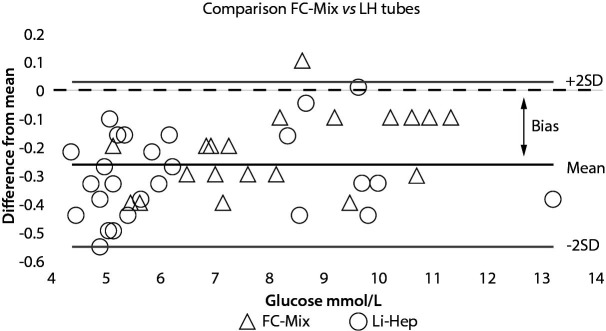
Differences in glucose results between Li-Heparin and Fluoride/Citrate tubes. Absolute differences of 49 paired samples, measured in Uppsala and Salzburg. SD – standard deviation. FC-Mix – Vacuette FC-Mix tube. Li-Hep – lithium heparin tube.

Since the second validation round showed differences between results from different tube additives, the most probable explanation for the differences between the two sites was the type of tube additive used for blood collection. In Uppsala a Na-F/C containing tube from GreinerBio-One was used (Vacuette FC-Mix, Greiner Bio-One, Kremsmünster, Austria) while in Salzburg a LH tube from Sarstedt (LH-Monovette, Sarstedt, Germany) was used. All other parts of the preanalytical procedures were identical. To confirm this hypothesis, another experiment was conducted at both sites. From routine clinical care patients (Salzburg N = 27, Uppsala N = 24), blood was collected into the mentioned Vacuette FC-Mix and LH-Monovette at the same time point and transported on slurry ice water to the laboratory, where the samples were centrifuged. Sample pairs were analysed in parallel to avoid any confounding bias on Roche COBAS instruments at both sites (Roche Diagnostics, Basel, Switzerland). After excluding two outliers, we observed a systematic bias between LH and Na-F/C tubes (Figure 2).

**Figure 3:**
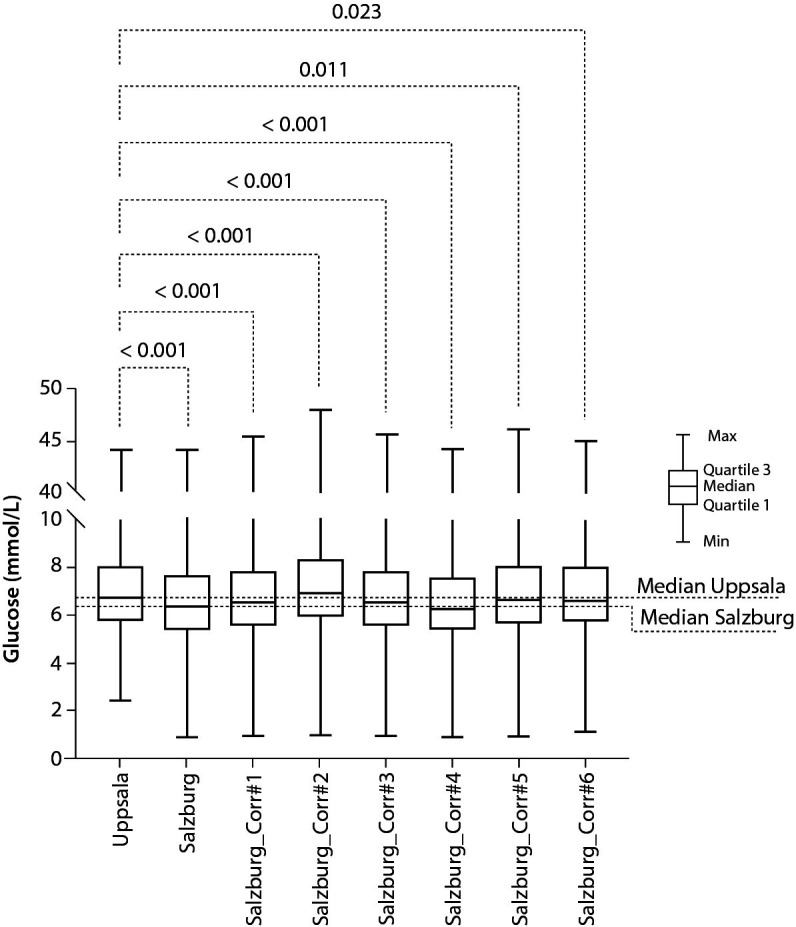
Glucose results of the study population with different correction factors applied. CF – correction factor. Comparison of the raw values collected at the two sites as well as adjusted results, applying CFs found in several studies comparing LH-tubes to NaF/C-tubes: #1 – Saracevic, A., *et al.* (*4*), CF = 1.0308; #2 - Bonetti, G., *et al.* (*1*), CF = 1.0886; #3 - van den Berg, S. A. A., *et al.* (*5*), CF = 1.0357; #4 - Bonettri, G., *et al.* (*2*); CF = 1.0018; #5 - Carey, R., *et al.* (*3*), CF = 1.0479; #6 – correction formula from our own experiment (Corrected Salzburg values = (1.0153 x Salzburg values) + 0.2489).

We then screened the literature and found several publications in support of our findings (*1*-*6*). We applied all of these biases and respective correction factors, including that from our own experiment, by application of the mean difference of sample pairs (Figure 2) to the results from Salzburg and Uppsala (Figure 3).

## What happened

Despite the profound and thorough harmonization of the study design, including a validation round for analytical comparability, the fact that the same tube was used for validating the analytical methods, but different tubes were used for the actual blood collection in the study subjects, was overlooked. This led to a systematic decrease of values in the Salzburg population since glycolysis is inhibited far better by Na-F/C than by LH (*6*).

## Discussion

In this article, we describe a serious preanalytical error that led to unnecessary follow-up investigations and eventually impaired validity of scientific data. While the same type of blood collection tube was used at both sites for validation of analytical results, LH tubes were used in Salzburg for blood collection in the study subjects. As the latter tubes do not inhibit glycolysis as sufficient as the combination of fluoride and citrate, results from Salzburg showed systematically lower values (*6*). When adjusting these values by applying correction factors from our own experiment as well as from published studies, values became far more comparable to those from the Uppsala population, which were measured from Na-F/C plasma. The fact that statistical tests still showed a difference in these adjusted values compared to the Uppsala results (P = 0.023), could indicate a biological difference in glucose tolerance between Uppsala and Salzburg subjects. This hypothesis would need further investigation.

Regulations on the use of certain glycolysis inhibiting blood collection tubes for glucose measurements differ between countries. Some recommend using Na-F/C tubes with citrate as immediate glycolysis inhibitor and Na-F as a long-term inhibitor if collection on ice-water slurry and centrifugation within 30 minutes cannot be guaranteed, while other countries have not applied similar regulations (*7*, *8*). Additionally, the use of tube type and vendor differs largely internationally, nationwide, and even within the same health care setting. In Austria, as an example, blood collection tubes from GreinerBioOne are widely used, while paediatric clinics tend to use the corresponding tubes from Sarstedt as suction during blood collection can be applied manually in these tubes, which is important especially in small or fragile veins. In Sweden, mostly tubes from BD are in use. As this vendor does not provide Na-F/C containing tubes, many health care settings use the FC-Mix tube from Greiner-BioOne instead.

These facts have some major implications. First, universally and internationally used cut offs for diagnosing impaired glucose tolerance (IGT) or diabetes might not fit to every setting (*9*, *10*). Second, comparing national registers regarding the glucose levels or the prevalence of Impaired Fasting Glucose (IFG), IGT, and diabetes mellitus type 2, according to the above-mentioned cut-offs might be invalid. Several studies have investigated differences in glucose levels and IGT prevalence between countries, either by comparing data from national registers or by comparing single studies against each other (*11*-*17*). However, in many cases, the important information on the preanalytical conditions, including the type of blood collection tubes used, was neglected, or not mentioned at all. Hagman *et al.* conducted such a comparison study between obese children and adolescents from Germany and Sweden and found mean fasting glucose levels of national registers at 4.6 (± 0.6) mmol/L and 5.0 (± 0.5) mmol/L, respectively (*14*). The authors conclude that there are marked differences between these countries for “unknown reasons”. The observed bias is very similar to the one found in our study populations and might therefore be attributed to the same root cause.

As these biases may trigger false categorization of patients and even overtreatment and surely unnecessary patient anxiety, the described preanalytical error might greatly jeopardize patient safety. Therefore, an obligatory international harmonization in preanalytical conditions for glucose level determination is urgently needed.

## What YOU can do in your study to prevent such errors

If you are conducting a study including any kind of laboratory testing, we highly recommend applying the PREDICT-checklist, provided by the European Federation of Clinical Chemistry and Laboratory Medicine (EFLM) Working Group for Preanalytical Phase (WG-PRE), to prevent any kind of preanalytical variable biasing your data (*18*).

When collecting blood for glucose determination, we recommend using Na-F/C tubes. These additives guarantee short-term and long-term inhibition of glycolysis, thereby securing a less altered glucose result.
